# Intravascular lymphoma masquerading as septic shock

**DOI:** 10.1002/ccr3.5656

**Published:** 2022-04-05

**Authors:** Hiroki Kato, Shinichi Miyazaki, Kodai Yabu, Yumi Sawada, Yuki Kondo, Tadashi Aoyama, Yoshiharu Nara, Motoyoshi Yano

**Affiliations:** ^1^ 37036 Department of Gastroenterology Yokkaichi Municipal Hospital Yokkaichi Japan; ^2^ 37036 Department of Respiratory Medicine Yokkaichi Municipal Hospital Yokkaichi Japan; ^3^ 37036 Department of Hematology Yokkaichi Municipal Hospital Yokkaichi Japan; ^4^ 37036 Department of Dermatology Yokkaichi Municipal Hospital Yokkaichi Japan; ^5^ 37036 Department of Anesthesiology and Critical Care Medicine Yokkaichi Municipal Hospital Yokkaichi Japan; ^6^ 37036 Department of Pathology Yokkaichi Municipal Hospital Yokkaichi Japan

**Keywords:** dyspnea, intravascular lymphoma, multiple‐organ dysfunction, septic shock

## Abstract

Intravascular lymphoma (IVL) is a rare type of extranodal lymphoma that selectively affects small blood vessels. We report a patient who presented with dyspnea and weight loss as well as refractory shock and multiple‐organ dysfunction. The postmortem revealed disseminated involvement of an IVL but no evidence of infection.

## Graphical abstract

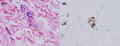

## 1 INTRODUCTION

Intravascular lymphoma is a rare extranodal subtype of diffuse large B‐cell lymphoma characterized by proliferation of lymphoma cells within the lumina of small blood vessels.^1^ The median age at diagnosis is 67 years, and there is no sex predilection.^2^, ^3^ The clinical presentation is diverse and often includes symptoms related to organ dysfunction caused by occlusion of blood vessels. We herein describe a patient who presented with refractory shock and multiple‐organ dysfunctions that mimicked septic shock. Postmortem examination revealed intravascular large B‐cell lymphoma and no evidence of infection.

## 2 CASE REPORT

**FIGURE 1:**
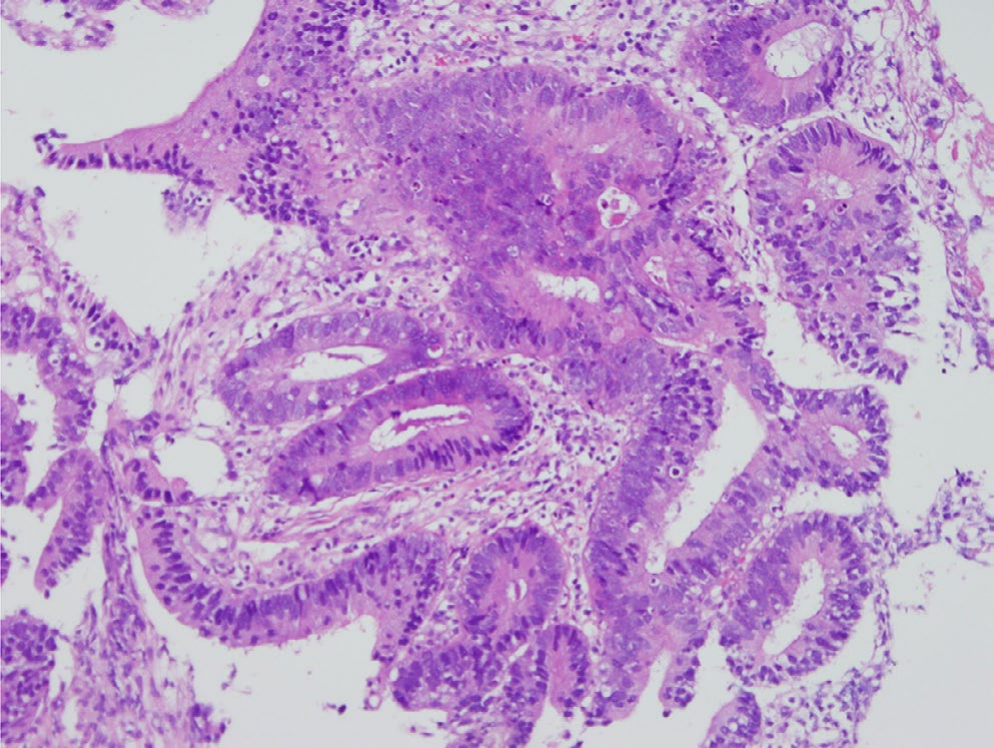
Colonic biopsy. Hematoxylin and eosin stain showing well‐differentiated adenocarcinoma

A previously healthy 64‐year‐old Japanese man presented to the emergency department with dyspnea on exertion, severe fatigue, decreased appetite, and weight loss for the preceding 2 weeks. One week before his presentation, esophagogastroduodenoscopy and colonoscopy were performed because of investigation of unintentional weight loss. The result revealed advanced rectal carcinoma (Figure 1). He did not report any chills, night sweats, or fevers, was a current cigarette smoker (44 pack‐year history of smoking), and reported minimal alcohol use. On examination, the patient appeared distressed, his respiratory rate was 30 breaths/min, oxygen saturation was 99% on oxygen supplementation at 2 L/min via nasal cannula, heart rate was 96 beats/min, blood pressure was 80/57 mm Hg, and body temperature was 35.8°C. He had conjunctival pallor, jaundice, and 4+ pitting edema in lower extremities, but no jugular venous distension, cardiac murmurs, or peripheral adenopathy.

**FIGURE 2:**
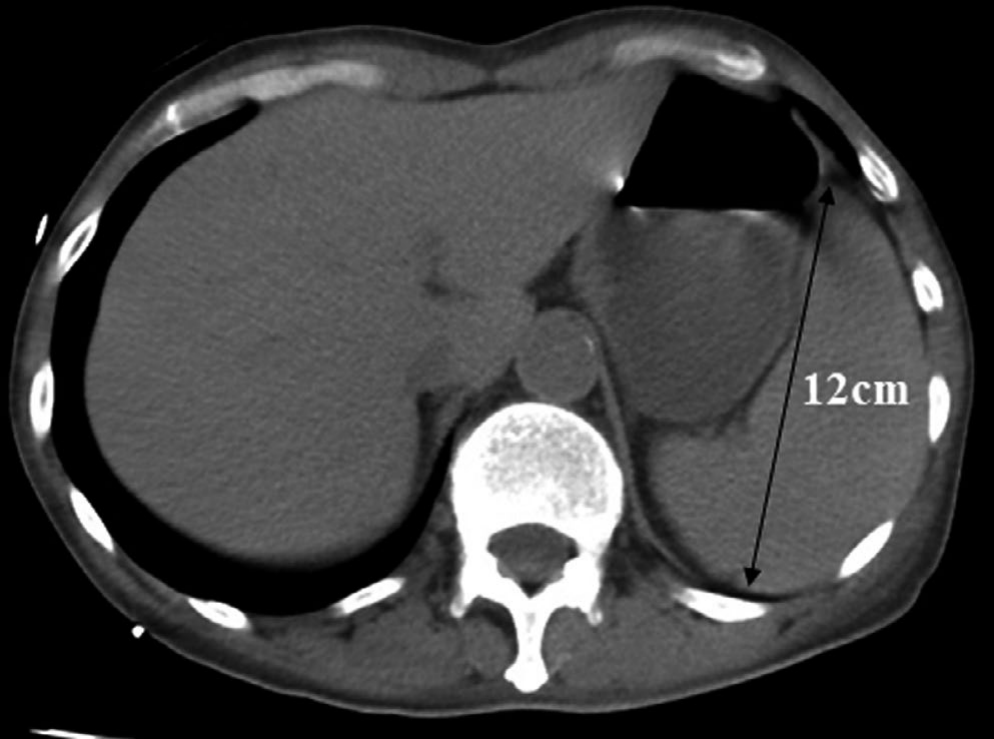
Computed tomography scan showing splenomegaly

Laboratory testing showed the following: white blood cell count = 21,000 cells/ml (neutrophils 76%, lymphocytes 16%, and monocytes 8%), hemoglobin = 9.5 g/dl, = platelet count = 65,000 cells/ml, International Normalized Ratio = 1.90, fibrinogen = 343 mg/dl, sodium = 131 mmol/L, potassium = 6.5 mmol/L, chloride = 96 mmol/L, blood urea nitrogen = 101 mg/dl, creatinine = 2.05 mg/dl, uric acid = 13.8 mg/dl, glucose = 67 mg/dl, aspartate aminotransferase = 38 U/L, alanine aminotransferase = 22 U/L, alkaline phosphatase = 113 U/L, lactate dehydrogenase (LDH) level = 478 U/L, total bilirubin = 3.0 mg/dl, albumin = 1.6 g/dl, total protein =4.9 g/dl, triglyceride = 251 mg/dl, ferritin = 900 ng/ml, soluble interleukin‐2‐receptor = 13,677 U/ml, procalcitonin = 3.6 ng/ml, C‐reactive protein (CRP) = 12 mg/dl, serum cortisol = 24.9 ug/dl (normal 4–18.3 ug/dl), and serum adrenocorticotrophic hormone = 15.6 pg/ml (normal 7.2–63.3 pg/ml). Arterial blood gas measurements showed a pH of 7.13, arterial partial pressure of carbon dioxide (PaCO_2_) of 9.9 mm Hg, and PaO_2_ of 156.0 mm Hg, with bicarbonate = 3.3 mmol/L and lactate =19.6 mmol/L. Blood cultures showed no growth. Urinalysis revealed 1+ protein and no blood leukocytes or bacteria. Chest radiographs and electrocardiogram were unremarkable. A computed tomography (CT) scan of the chest, abdomen, and pelvis revealed only splenomegaly (Figure 2).

**FIGURE 3:**
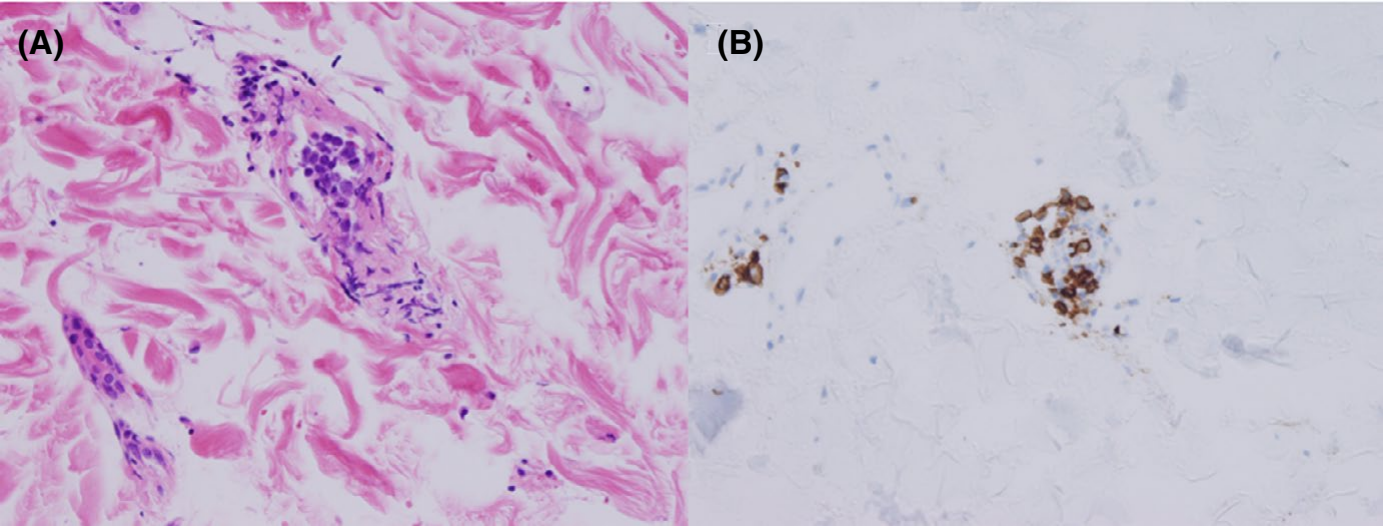
Random skin biopsy. Hematoxylin and eosin stain showing malignant cells within small‐vessel lumen (A). CD20 stain (brown) showing malignant cells that are CD20‐positive (B)

**FIGURE 4:**
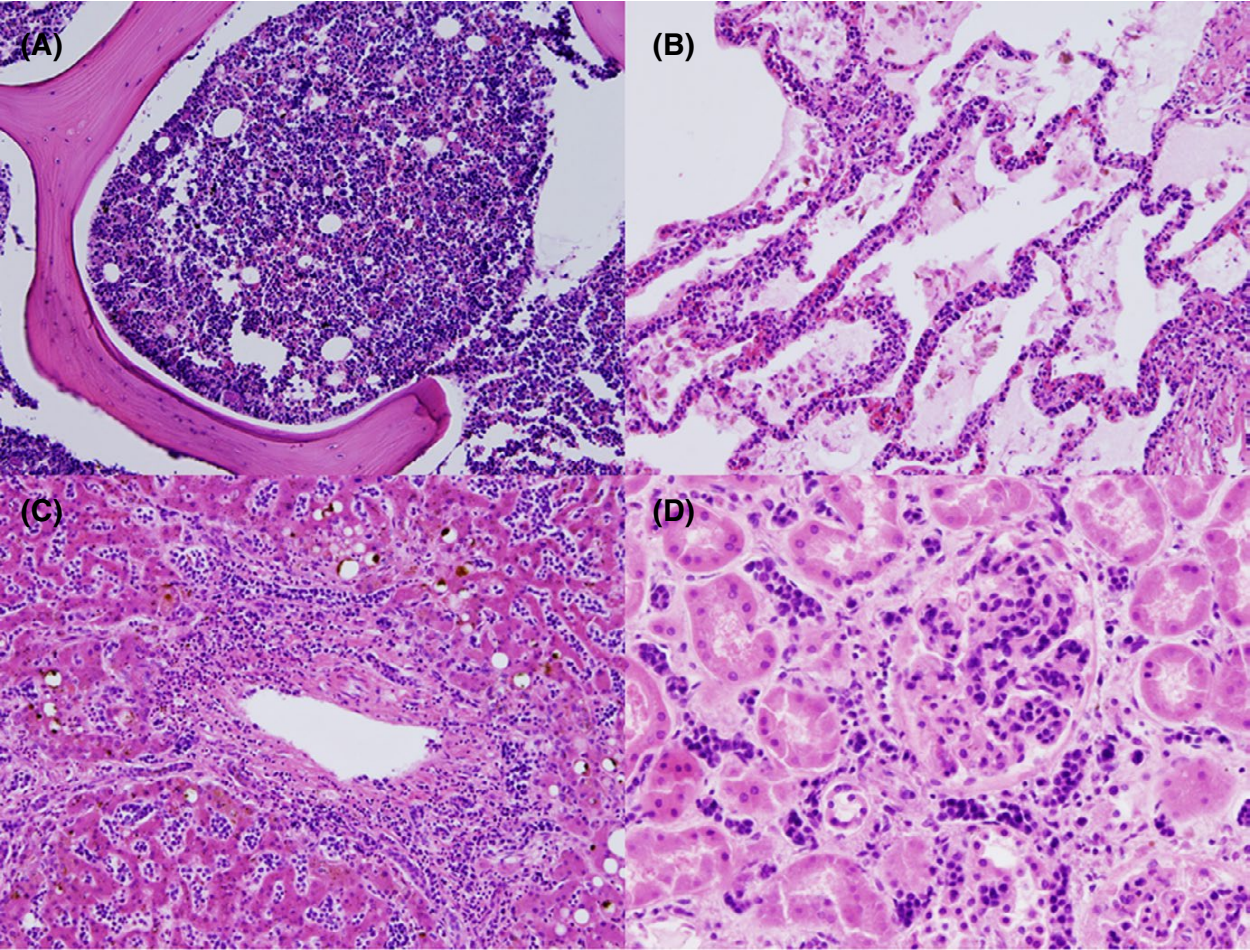
Hematoxylin and eosin staining of postmortem bone marrow (A), lung (B), liver (C), and kidney (D) biopsies revealed atypical lymphoid cells within small‐vessel lumen

**FIGURE 5:**
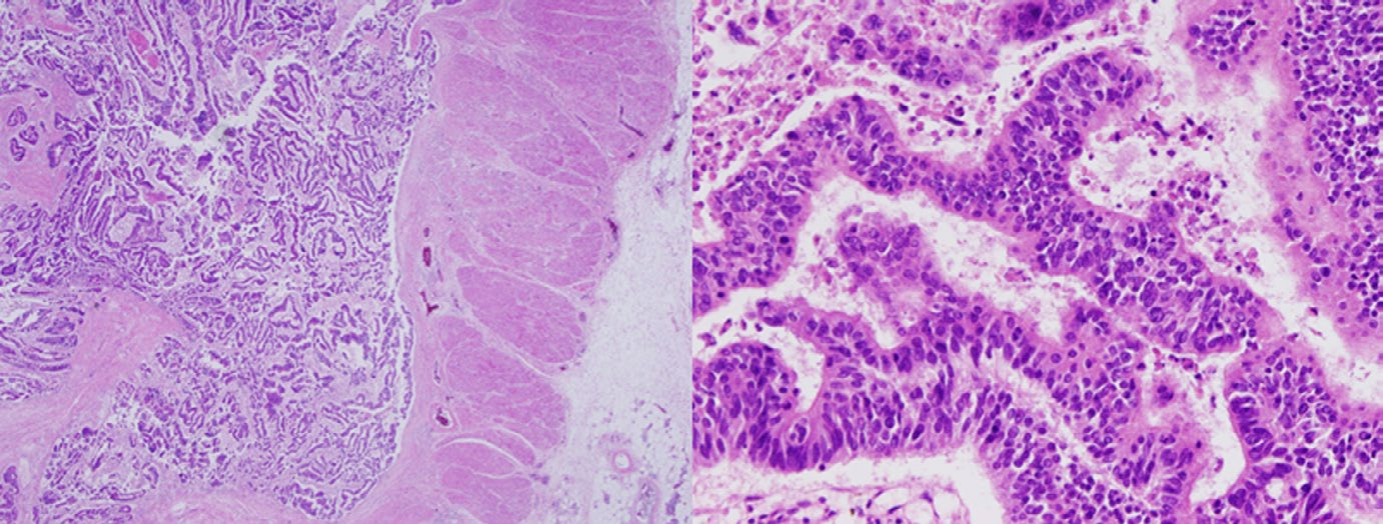
Postmortem rectal biopsy. Hematoxylin and eosin stain showing rectal carcinoma confined to invasion of the muscularis propria

Despite aggressive fluid resuscitation, the patient remained persistently hypotensive and was admitted to the intensive care unit. A diagnosis of septic shock was considered, and the patient was started on vasopressors and empirical broad‐spectrum antibiotics. The clinical presentation of pancytopenia, splenomegaly, and severe lactic acidosis, without lymphadenopathy, was consistent with hematologic malignancy, especially intravascular lymphoma. In addition to bone marrow biopsy, a random skin biopsy was performed. While awaiting pathological diagnosis, the patient went into shock and experienced multiple‐organ dysfunction syndrome involving the circulatory system, liver, and coagulation system (Table 1). He died 5 days after hospitalization. Later, biopsies from the bone marrow and skin revealed intravascular large B‐cell lymphoma (Figure 3). A postmortem examination was notable for the demonstration of intravascular large B‐cell lymphoma with extensive involvement of the bone marrow, lungs, liver, spleen, kidneys, and adrenals (Figure 4). The rectal carcinoma was confined to invasion of the muscularis propria (Figure 5), and no lymph node or distant metastasis was detected. No infectious focus was found.

**TABLE 1:** Laboratory data

Laboratory	Admission	Day 2	Day 3	Day 4	Day 5	Day 6
WBC count, K/ml	21	11.6	8.8	7.1	9.6	9.9
Hemoglobin, g/dl	9.5	8.1	8.7	7.1	9.3	9.2
Platelet count, K/ml	6.5	4.7	2.2	1.2	2.3	2.0
Fibrinogen, mg/dl	343	241	212	206	235	233
INR	1.90	1.61	1.45	1.27	1.35	1.61
D‐dimer, μg/ml			7.1	7.1	9.0	8.7
pH	7.129	7.463	7.463	7.401	7.382	7.250
Bicarbonate, mmol/L	3.3	15.0	18.6	17.9	14.4	7.6
Lactate, mmol/L	19.6	11.7	12.1	13.4	18.3	26.5
Total bilirubin, mg/dl	3.0	3.8	6.2	12.3	19.6	24.9
Conjugated bilirubin, mg/dl		2.5	4.5	9.7	15.8	21.8
ALT, U/L	22	19	19	18	19	20
ALP, U/L	113	89	102	96	146	208
LDH, U/L	478	426	465	446	627	845
BUN, mg/dl	101.0	97.8	76.3	65.6	67.9	75.9
Serum creatinine, mg/dl	2.05	1.69	1.13	0.98	0.99	1.18

## 3 DISCUSSION

The patient was a 64‐year‐old man with a 2‐week history of dyspnea on exertion and weight loss. Key findings included elevated CRP level, refractory shock, pancytopenia, splenomegaly, and lactic acidosis. Despite treatment for presumed septic shock, rapid progression of multiple‐organ dysfunction developed, and the patient died 5 days after hospitalization. Postmortem examination revealed evidence of intravascular large B‐cell lymphoma, and no infectious focus.

Intravascular lymphoma (IVL) was first described by Pleger and Tapeiner in 1959 as angioendotheliomatosis proliferans systemisata^4^ and was initially thought to be a neoplasm of endothelial origin, but subsequent immunohistochemical studies confirmed the lymphoid nature of the neoplasm.^5^ Almost all reported cases of IVL have been a neoplasm of B cells.^6^ IVL characteristically manifests as fever, pronounced fatigue, decreased appetite, and a decline in functional status.^3^ The clinical presentation is diverse and appears to differ by country of origin.^7^, ^8^ In Western countries, patients present most commonly with symptoms related to involvement of the central nervous system (39%) and skin (40%). Bone marrow (32%), liver (26%), and spleen (26%) involvement are less common. Patients from Asian countries preferentially show bone marrow involvement (75%), hepatomegaly (55%), splenomegaly (67%), and hemophagocytic syndrome (61%). Less commonly involved tissues include the nervous system (27%) and skin (15%). Patients often present with the following laboratory abnormalities^9^: anemia (93%), leukopenia (62%), and thrombocytopenia (51%) as well as elevated of serum LDH (97%), CRP (96%), and ferritin (83%). Patients from Western countries and Asian countries differ in the rates of hypoalbuminemia (18% vs. 84%).

The patient's initial presentation was consistent with septic shock, including elevated CRP level, thrombocytopenia, hyperbilirubinemia, renal insufficiency, and refractory shock.^10^ Neither microbiology nor autopsy confirmed an infectious source that would account for the clinical picture. Shock is an unusual initial presentation of IVL and has been previously described only in case reports (11–15, Table 2).

**TABLE 2:** Clinical features of intravascular lymphoma with shock

	Ip M^11^	Deusch E^12^	Yalamanchili M^13^	Fenot M^14^	Srivali N^15^	This case
Age/Sex	28/M	53/F	62/M	56/F	82/M	64/M
Symptom/Sign	Fever, rash, splenomegaly, polyneuropathy	Fever, splenomegaly, hydronephrosis	Fever	Fever, dyspnea	Anasarca	Dyspnea, splenomegaly, anasarca
Organ involvement	Brain, lungs, liver, spleen, kidneys, lymph nodes, ileum, and prostate at autopsy	Thyroid, lungs, liver, kidneys, adrenals at autopsy	Bone marrow, lung, liver, spleen, kidney, small bowel, colon, and bladder at autopsy	Skin	Skin	Lungs, liver, spleen, kidneys, and adrenals at autopsy
Outcome	Death	Death	Death	Death	Death	Death
Survival from initial onset to death	2 months	5 months	13 Days	Not available	1 month	19 Days

Diagnosis of IVL requires a tissue biopsy of an involved organ. Although bone marrow, lymph nodes, peripheral blood, and cerebrospinal fluid are often uninvolved in IVL,^16^ the most relevant diagnostic site seems to be the bone marrow in Asian cohorts.^8^ Furthermore, random biopsy of apparently normal skin is a valuable, relatively noninvasive, diagnostic tool.^17^ The fact that lymphoma cells have been identified from random skin biopsies, not only in European series in which the cutaneous involvement is prevalent, but also in Asian series in which the cutaneous involvement is rare, is remarkable.^18^, ^19^

This case shows that IVL can be a cause of refractory shock of unknown origin. The patient underwent a prompt evaluation over a period of days to investigate his pancytopenia and splenomegaly. Unfortunately, the antemortem diagnosis of IVL could not be established. Prior to diagnostic biopsy, corticosteroid treatment complicates the histological interpretation and may delay definitive diagnosis.^20^ However, after the biopsy, corticosteroid therapy can be tried and may improve critical illness transiently but dramatically, without a diagnostic problem.^21^

## 4 CONCLUSION

In the first 24 h of management of suspected septic shock, the absence of a source of infection is not so uncommon, and the septic focus may be identified later. If no source is found, reassess the differential diagnosis and consider many mimics for sepsis. This case highlights that intravascular lymphoma can be a cause of refractory shock of unknown origin.

## CONFLICT OF INTEREST

The authors declare that they have no conflicts of interest.

## AUTHOR CONTRIBUTIONS

Hiroki Kato and Shinichi Miyazaki involved in conception and design of study, literature search, and drafting of article. Kodai Yabu, Yumi Sawada, Tadashi Aoyama, and Yoshiharu Nara involved in data acquisition and interpretation. Yuki Kondo and Motoyoshi Yano involved in proofreading the manuscript. All authors read and approved the final manuscript.

## ETHICAL APPROVAL

None.

## CONSENT

Written informed consent was obtained from the kin to publish this report in accordance with the journal's patient consent policy.

## Data Availability

None.
